# Modified bladder outlet obstruction index for powerful efficacy prediction of transurethral resection of prostate with benign prostatic hyperplasia

**DOI:** 10.1186/s12894-021-00937-x

**Published:** 2021-12-06

**Authors:** Hongming Liu, Ye Tian, Guangheng Luo, Zhiyong Su, Yong Ban, Zhen Wang, Zhaolin Sun

**Affiliations:** 1 Department of Urology, The Affiliated Hospital of Guizhou Medical University, Guiyang, China; 2 Department of Urology, Guizhou Provincial People’s Hospital, Guiyang, China

**Keywords:** Benign prostatic hyperplasia, Transurethral resection of prostate, Modified bladder outlet obstruction index, Surgical efficacy

## Abstract

**Background:**

The correlation between modified bladder outlet obstruction index (MBOOI) and surgical efficacy still remains unknown. The purpose of the study was to investigate the clinical value of the MBOOI and its use in predicting surgical efficacy in men receiving transurethral resection of the prostate (TURP).

**Methods:**

A total of 403 patients with benign prostate hyperplasia (BPH) were included in this study. The International Prostate Symptom Score (IPSS), quality of life (QoL) index, transrectal ultrasonography, and pressure flow study were conducted for all patients. The bladder outlet obstruction index (BOOI) (P_det_Q_max_–2Q_max_) and MBOOI (P_ves_–2Q_max_) were calculated. All patients underwent TURP, and surgical efficacy was accessed by the improvements in IPSS, QoL, and Q_max_ 6 months after surgery. The association between surgical efficacy and baseline factors was statistically analyzed.

**Results:**

A comparison of effective and ineffective groups based on the overall efficacy showed that significant differences were observed in PSA, P_ves_, P_det_Q_max_, P_abd_, BOOI, MBOOI, TZV, TZI, IPSS-t, IPSS-v, IPSS-s, Q_max_, and PVR at baseline (*p* < 0.05). Binary logistic regression analysis suggested that MBOOI was the only baseline parameter correlated with the improvements in IPSS, QoL, Q_max_, and the overall efficacy. Additionally, the ROC analysis further verified that MBOOI was more optimal than BOOI, TZV and TZI in predicting the surgical efficacy.

**Conclusion:**

Although both MBOOI and BOOI can predict the clinical symptoms and surgical efficacy of BPH patients to a certain extent, however, compared to BOOI, MBOOI may be a more useful factor that can be used to predict the surgical efficacy of TURP.

*Trial registration* retrospectively registered.

## Background

Benign prostatic hyperplasia (BPH), whose prevalence progressively increases with age, is one of the most common diseases in middle-aged and elderly men [1]. Currently, static and dynamic obstruction due to benign prostatic enlargement (BPE) or/and benign prostatic obstruction (BPO) is considered as the main cause of low urinary tract symptom (LUTS), which has a severe impact on the physical and mental health and quality of life (QoL) of elderly men.

Pressure-flow studies (PFSs) have been recommended as the gold standard for diagnosing bladder outlet obstruction (BOO) by the International Continence Society, among which the BOO index (BOOI) has become best-known and most widely-adopted urodynamic parameter [2, 3]. It is routinely used to evaluate the condition of BPH patients and gauge the efficacy of corrective surgery. Nevertheless, in our previous study, it was observed there was no significant correlation between BOOI and symptoms and the maximum urine flow rate (Q_max_) in BPH patients [4]. In fact, as the resistance to urination increases with the progression of BOO, many patients undergo abdominal straining to urinate during a PFS. The process of urination involves both detrusor pressure and abdominal pressure, and it is obviously insufficient to only consider the detrusor pressure. Therefore, research has been carried out to assess the correlation between abdominal pressure and BOO, and it has been previously determined that a modified BOOI (MBOOI) that takes into account abdominal pressure can better predict the BOO than the BOOI [5].

The treatments of LUTS secondary to BPH include drug treatment and surgical treatment, among which transurethral resection of prostate (TURP) is still regarded by the current guidelines as the gold standard for surgical treatment [6]. Although TURP is recognized as a safe and effective treatment, significant efficacy is not achieved for all patients. Surgical failure is more likely to occur in patients with detrusor dysfunction and lower baseline BOOI [7]. It has also been found that the degree of preoperative BOO is positively associated with improvement in LUTS and QoL after TURP [8]. Therefore, a preoperative PFS is recommended for optimal selection of patients who are more suitable for surgery by measuring BOOI and assessing detrusor function.

As mentioned above, BOOI (P_det_Q_max_–2Q_max_) does not consider the role of abdominal straining in urination, or a predicted BOO may be worse than a MBOOI. Additionally, the correlation between MBOOI and surgical efficacy still remains unknown. Hence, we hypothesized that MBOOI predicts the surgical outcome more optimally than BOOI, and thus, the purpose of this study was to assess the value of MBOOI in predicting the surgical efficacy of TURP by comparing it with BOOI and other parameters.

## Methods

### Patient cohort

This was a retrospective study that received approval by the Hospital Ethics Committee of GuiZhou Provincial People’s Hospital, and written informed consent was obtained (No. 2018054). From November 2015 to March 2020, a total of 403 patients with LUTS/BPH were enrolled in the study. In addition to routine examination, such as digital rectal examination, serum prostate-specific antigen (PSA), and kidney-bladder ultrasound, the International Prostate Symptom Score (IPSS), transrectal ultrasonography (TRUS), and PFS were routinely performed before surgery, otherwise, the patients were not included in the study. The non-inclusion criteria included: (1) bladder calculi, bladder tumor, neurogenic bladder dysfunction, urethral stricture, and other diseases that may affect the function of urination; (2) previous surgery of the prostate and/or bladder and/or urethra; (3) prostate cancer that was confirmed by postoperative pathology. The patients with suspected prostate cancer underwent an ultrasound-guided transrectal prostate biopsy for confirmation or exclusion of cancer. The indications for the operation are as follows: recurrent or refractory urinary retention, overflow incontinence, recurrent urinary tract infections, bladder stones or diverticula, treatment-resistant macroscopic haematuria due to BPH/BPE, or dilatation of the upper urinary tract due to BPO(with or without renal insufficiency), insufficient relief of LUTS after conservative or medical treatments [6]. All patients were followed up and reassessed with IPSS, QoL, and free flowmetry 6 months later.

### Assessment of prostatic anatomical parameters

TRUS (Philips EPIQ 5) was used to estimate the total prostate volume (TPV) and transitional zone volume (TZV) by the prostate ellipsoid formula (height × width × length × π/6). The transitional zone index (TZI) was calculated by TPV and TZV (TZI = TZV/TPV) [9].

### Assessment of urinary symptoms and urodynamic measurements

Subjective symptoms were assessed by the IPSS and QoL questionnaires, including IPSS total score (IPSS-t), IPSS voiding score (IPSS-v), IPSS storage score (IPSS-s), post-micturitional IPSS score (IPSS-p), and QoL score. A PFS was performed by multichannel urodynamic evaluation (UDS-94-BT, Delphis, Laborie, Canada) to assess objective symptoms. An 8-F double-lumen catheter was transurethrally inserted, and a 10-F single-lumen catheter was transrectally inserted with the patient in a sitting position. The bladder was perfused with physiological saline solution (20–50 ml/min) until the patient felt a strong desire to urinate (maximum bladder volume), bladder perfusion was then stopped, and the patient was ordered to urinate into the collector. Maximum bladder volume, intra-vesical pressure (P_ves_), abdominal pressure (P_abd_), Q_max_, and post void residual (PVR) urine volume were simultaneously measured. Detrusor pressure at maximum urine flow rate (P_det_Q_max)_ is equal to P_ves_ minus P_abd_, and the BOOI (P_det_Q_max_–2Qmax) and MBOOI (P_ves_–2Q_max_) were calculated by P_ves_, P_det_Q_max_, and Q_max_ [10].

### Assessment of surgical efficacy of TURP

Surgical efficacy was determined according to the improvement of IPSS, QoL score, and Q_max_ after surgery. The degree of improvement was judged as poor (level 1), fair (level 2), good (level 3), and excellent (level 4). IPSS improvement > 75% was considered excellent, 50–75% good, 25–50% fair, and ≤ 25% none. A QoL improvement of 4–6 score was classified excellent, 3 score good, 1–2 score fair, and 0 score poor. A Q_max_ improvement ≥ 10.0 ml/s was considered excellent, 5.0–10.0 ml/s good, 2.5–5.0 ml/s fair, and < 2.5 ml/s poor. The median of the three aspects (IPSS, QoL score, and Q_max_) was defined as the overall efficacy level, in which levels 3 and 4 were defined as effective, and levels 1 and 2 as ineffective (Table 1) [11].

**Table 1:** Surgical efficacy based on the improvements in symptoms, QoL and function

Efficacy grade	Criteria	No. patients (%)
*Symptom: Post/pre ratio of IPSS-t*		
Excellent	≤ 0.25	161 (39.95)
Good	≤ 0.50	146 (36.23)
Fair	≤ 0.75	69 (17.12)
Poor	> 0.75	27 (6.70)
*QoL: Pre-post of QoL index*		
Excellent	6,5,4	139 (34.49)
Good	3	125 (31.02)
Fair	2,1	105 (26.05)
Poor	0	34 (8.44)
*Function: Post–pre of Q*_*max*_		
Excellent	≥ 10 mL/s	116 (28.78)
Good	≥ 5 mL/s	172 (42.68)
Fair	≥ 2.5 mL/s	77 (19.11)
Poor	< 2.5 mL/s	38 (9.43)
*The overall efficacy: median of efficacy grades of symptom, function and QoL*		
Excellent		136 (33.75)
Good		161 (39.95)
Fair		84 (20.84)
Poor		22 (5.46)

IPSS-t = international prostate symptom total score, QoL = quality of life, Q_max_ = maximum urine flow rate

### Statistical analysis

All statistical values were reported as the mean ± standard deviation. Kolmogorov–Smirnov test was used to determine whether the continuous variables were in line with normal distribution. If the variables were normally distributed, Student’s t-test was applied to compare difference in preoperative factors between two groups according to the overall efficacy. The non-normal distribution variables were conducted with Mann–Whitney U test. Simple linear regression analysis was applied to determine the significant predicting factors for therapeutic effects, and then, stepwise forward binary logistic regression analysis was carried out to determine the factors associated with surgical outcomes of TURP. The receiver operating characteristic (ROC) curves were produced, and the area under the curve (AUC) was subsequently calculated to describe the predictive value of MBOOI in surgical outcomes. All Statistical analysis were processed using IBM SPSS 25.0 for Windows statistical software (Statistical Package for Social Sciences, IBM Corporation, Armonk, NY, USA). All statistical tests were two-sided, *p* < 0.05 was considered to be statistically significant.

## Results

### Comparison of baseline characteristics between the effective and ineffective groups based on the overall efficacy

A total of 403 patients between 53–90 years of age diagnosed with BPH were included in the present study. The general characteristics of the study population are shown in Table 2. The surgical efficacy rates according to the improvements in IPSS, QoL, and Q_max_ after surgery were 76.18%, 65.51%, and 71.46% respectively, and the overall efficacy rate of TURP was 73.70%. Kolmogorov–Smirnov test results showed that Q_max_ followed normal distribution, therefore, Student’s *t*-test was applied to compare difference in Q_max_ between two groups according to the overall efficacy. Mann–Whitney U test was applied to compare difference in other preoperative factors between two groups due to these variables in accordance with normal distribution. A comparison of the overall efficacy in the effective and ineffective groups revealed significant differences in PSA (*p* = 0.021), P_ves_ (*p* < 0.001), P_det_Q_max_ (*p* < 0.001), P_abd_ (*p* < 0.001), BOOI (*p* < 0.001), MBOOI (*p* < 0.001), TZV (*p* = 0.022), TZI (*p* = 0.025), IPSS-t (*p* < 0.001), IPSS-v (*p* = 0.014), IPSS-s (*p* < 0.001), Q_max_ (*p* = 0.010), and PVR (*p* = 0.006) at baseline, but significant differences were not observed in age (*p* = 0.105), TPV (*p* = 0.074), IPPS-p (*p* = 0.520), or QoL (*p* = 0.357) (Table 2).

**Table 2:** Baseline clinical characteristics and comparison of preoperative characteristics between the two groups classified by the overall surgical efficacy

Variables	Baseline (n = 403)	Effective (n = 297)	Ineffective (n = 106)	*p* Value
Age (year)	70.94 ± 7.50	71.30 ± 7.44	69.94 ± 7.62	0.105
PSA (umol/L)	4.86 ± 4.98	5.21 ± 5.30	3.88 ± 3.83	0.021
*Ultrasonography*				
TPV (mL)	52.84 ± 27.63	54.19 ± 28.06	49.05 ± 26.12	0.074
TZV (mL)	24.90 ± 19.45	26.09 ± 19.92	21.59 ± 17.73	0.022
TZI	0.43 ± 0.14	0.44 ± 0.15	0.40 ± 0.12	0.025
*Urodynamics*				
P_ves_ (cmH_2_O)	102.72 ± 40.04	110.38 ± 40.58	81.23 ± 29.38	< 0.001
P_det_Q_max_(cmH_2_O)	84.92 ± 35.23	90.57 ± 36.07	69.10 ± 27.22	< 0.001
P_abd_ (cmH_2_O)	17.79 ± 16.40	19.81 ± 17.98	12.13 ± 8.65	< 0.001
Q_max_(mL/s)	8.10 ± 3.37	7.84 ± 3.31	8.82 ± 3.43	0.010
BOOI	68.73 ± 35.66	74.89 ± 36.41	51.47 ± 26.90	< 0.001
MBOOI	86.52 ± 40.59	94.71 ± 41.04	63.59 ± 28.96	< 0.001
PVR (mL)	74.36 ± 78.10	79.54 ± 78.75	59.85 ± 74.73	0.006
*International prostate symptom score (IPSS)*				
IPSS-t	22.51 ± 5.22	23.10 ± 5.17	20.84 ± 5.03	< 0.001
IPSS-v	8.72 ± 3.45	8.99 ± 3.46	7.98 ± 3.34	0.014
IPSS-s	10.25 ± 2.71	10.61 ± 2.65	9.24 ± 2.61	< 0.001
IPSS-p	3.51 ± 1.54	3.48 ± 1.54	3.59 ± 1.54	0.520
IPSS QoL	4.73 ± 1.03	4.77 ± 0.99	4.62 ± 1.14	0.357

PSA = prostate-specific antigen, TPV = total prostate volume, TZV = transitional zone volume, TZI = transitional zone index, P_ves_ = intra-vesical pressure, P_det_Q_max_ = detrusor pressure at maximum urine flow rate, P_abd_ = abdominal pressure, Q_max_ = maximum urine flow rate, BOOI = bladder outlet obstruction index, MBOOI = modified BOOI, PVR = post void residual urine volume, IPSS-t = IPSS total score, IPSS-v = IPSS voiding score, IPSS-s = IPSS storage score, IPSS-p = post-micturitional IPSS score, QoL = quality of life

### Association of surgical efficacy with preoperative variables

As presented in Table 3, simple linear regression analysis was used to analyze the correlations between preoperative factors and the surgical efficacy in IPSS, QoL, and Q_max_. All preoperative variables that were significantly correlated with surgical efficacy in IPSS, QoL, and Q_max_ using simple linear regression analysis were analyzed by stepwise forward binary logistic regression. From the results, MBOOI (*p* < 0.001) and IPSS-t (*p* < 0.001) were correlated with improvement of IPSS-t (*p* < 0.05). MBOOI (*p* < 0.001), P_abd_ (*p* = 0.035) and QoL (*p* < 0.001) with improvement of QoL. Meanwhile, MBOOI (*p* < 0.001) and Q_max_ (*p* < 0.001) with improvement of Q_max_. In addition, improved MBOOI (*p* < 0.001) and IPSS-t (*p* = 0.009) were correlated with the overall efficacy of TURP. Particularly, MBOOI was the only preoperative factor correlated with the surgical efficacy in IPSS, QoL, Q_max_, and the overall both (Table 3).

**Table 3:** Relationship between the baseline factors and surgical efficacy in IPSS-t, Q_max_, QoL and the overall surgical efficacy in binary logistic regression

Variables	OR	(95% CI)	*p* Value
*Surgical efficacy in IPSS-t*			
MBOOI	1.021	(1.012–1.029)	< 0.001
IPSS-t	1.101	(1.049–1.156)	< 0.001
*Surgical efficacy in QoL index*			
P_abd_ (cmH_2_O)	1.022	(1.001–1.043)	0.035
MBOOI	1.021	(1.013–1.030)	< 0.001
QoL	1.962	(1.541–2.498)	< 0.001
*Surgical efficacy in Q*_*max*_			
MBOOI	1.026	(1.018–1.035)	< 0.001
Qmax (mL/s)	0.793	(0.733–0.857)	< 0.001
*The overall surgical efficacy*			
MBOOI	1.027	(1.018–1.036)	< 0.001
IPSS-t	1.064	(1.016–1.115)	0.009

**Fig. 1:**
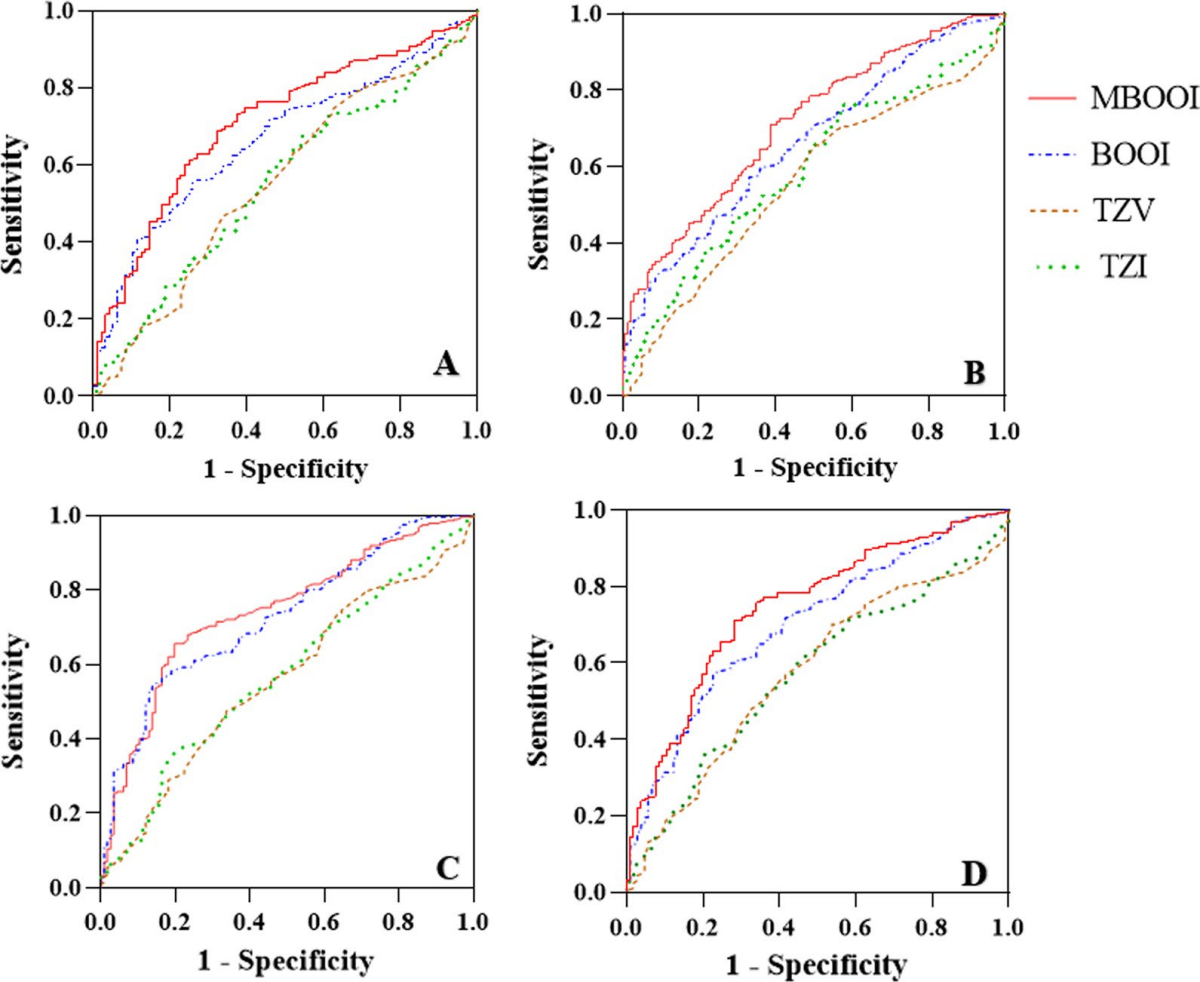
ROC curve analysis comparing MBOOI, BOOI, TZV and TZI in predict surgical efficacy. TURP efficacy in IPSS-t (**A**). TURP efficacy in QoL (**B**). TURP efficacy in Q_max_ (**C**). The overall surgical efficacy **(D)**

Furthermore, as shown in Fig. 1, the ROC curve was plotted, and the AUC was calculated. ROC analysis further demonstrated that MBOOI (AUC = 0.744, 95% CI 0.691–0.798) was more optimal than BOOI (AUC = 0.701, 95% CI 0.645–0.757), TZV (AUC = 0.575, 95% CI 0.513–0.636), and TZI (AUC = 0.573, 95% CI 0.513–0.634) in predicting the overall surgical efficacy of TURP. With a larger AUC, there was a higher correlation of MBOOI (AUC = 0.708, 95% CI 0.652–0.765) with the improvement in IPSS-t than BOOI (AUC = 0.664, 95% CI 0.606–0.721), TZV (AUC = 0.556, 95% CI 0.491–0622), and TZI (AUC = 0.543, 95% CI 0.484–0.618). Similarly, compared with BOOI (AUC = 0.661, 95% CI 0.608–0.715), TZV (AUC = 0.558, 95% CI 0.501–0.616), and TZI (AUC = 0.582, 95% CI 0.252–0.639), MBOOI (AUC = 0.710, 95% CI 0.659–0.761) had a larger AUC in improvement in QoL. With regard to the surgical efficacy in Q_max_, the AUC was 0.742 (95% CI 0.691–0.794) for MBOOI, 0.728 (95% CI 0.676–0.779) for BOOI, 0.559 (95% CI 0.499–0.619) for TZV, and 0.570 (95% CI 0.510–0.630) for TZI (Fig. 1).

## Discussion

BOO is one of the main causes of LUTS. IPSS is currently recognized as the most effective method to evaluate the severity of subjective symptoms in BPH patients, and PFS is an objective examination to quantify the condition as well as pre-surgical and post-surgical efficacy. The degree of BOO is classified into three grades by BOOI: unobstructed (BOOI ≤ 20), equivocal (20 < BOOI ≤ 40), and obstructed (BOOI > 40) [12]. However, Han et al. noted that BOOI is often inconsistent with endoscopically proven obstruction due to exclusion of the role of abdominal pressure in urination, and thus, they proposed the concept of modified BOOI and proved that modified BOOI can better predict BOO in patients with LUTS/BPH [5]. This finding is consistent with the results of our study, where MBOOI, when compared with BOOI, exhibited a higher correlation not only with IPSS, QoL, and Q_max_, but also with PSA, TPV, and TZI.

TURP is the standard surgical method for the treatment of BPH in prostate volume ≤ 80 ml. With the improvement of surgical proficiency and technology, TURP is also commonly used in patients with larger prostate, and it is equally safe and effective in large size prostate (> 80 ml) as compare in small size (≤ 80 ml) [6, 13, 14]. TURP is not only the mainstream surgical method at present, but also often the preferred surgical method for BPH. Therefore, in this study, we did not limit the volume of prostate in the included patients. The mean prostate volume (52.84 ± 27.63) in this study was small, but this is consistent with the results of related studies, the mean PV in each 10- year age group was lower that reported in studies in Asian populations than that in studies on Caucasians and African Americans [15–17]. However, further studies are needed to clarify whether the same results can be obtained from a separate study of large prostate patients, especially prostate volume larger than 80 ml. This study mainly focuses on the prediction of TURP efficacy by MBOOI. Nonetheless, 5–35% of patients postoperatively report persistent symptoms after TURP [18]. Therefore, in clinical practice, it is highly necessary to predict whether invasive surgery will be beneficial for patients. Traditionally, to select appropriate patients for surgery, BOOI with a PFS is recommended. In a study by Seki et al., multiple logistic regression analysis indicated that a higher baseline level of BOOI was associated with greater improvements in IPSS and QoL. Huang et al. conducted a study to establish an efficacy prediction model for transurethral prostatectomy, and found that there was a positive correlation between surgical efficacy with a higher degree of BOO and secondary detrusor cell hypertrophy [19]. Similarly, previous studies have shown that patients with definite BOO derive greater benefit from TURP surgery than those with equivocal and unobstruction [20, 21]. Previous studies indicated that BOOI is an extremely important method for predicting the surgical outcome of TURP.

The limitations of BOOI are emerging. Han et al. followed up 71 patients from 12 to 55 months, and found that 64% of patients were satisfied with the surgical results in the unobstructed and weak bladder contractility group [22]. Although the surgical effect in the BOO-positive group was significantly better than that of those in the BOO-negative group, Kim indicated that being BOO-positive might not be the absolute surgical indication for TURP [23]. Han et al. found that abdominal pressure was correlated with the degree of BOO as defined by cystourethroscopy [5]. In our study, abdominal pressure was a predictive factor for improvement of QoL. Sekido stated that abdominal pressure serves as a compensatory mechanism to promote urination with impaired detrusor and bladder contractions, and an increase in abdominal pressure would reduce the detrusor pressure required to achieve the same flow rate [24]. Consequently, P_det_Q_max_, which is obtained by subtracting the P_abd_ from P_ves_ for analysis of pressure flow, may lead to a vague interpretation of the P-Q diagrams and an incorrect assessment of outflow impedance [25]. However, the specific mechanism governing abdominal straining in urination remains unknown.

Therefore, in order to better evaluate patients' conditions and predict surgical efficacy, it is vital to determine more valuable parameters that take into account abdominal pressure. Here, we compared the results of a simple modified method with the traditional BOOI, and the present findings confirm that MBOOI appears to be better at predicting surgical outcomes than BOOI. In one respect, MBOOI, providing clearly better results than BOOI, was significantly related to the changes in IPSS (including IPSS-v, IPSS-s, IPSS-p, IPSS-t), QoL, Q_max_, and PVR after TURP. However, superior results were observed for the association between MBOOI and surgical outcomes, which were accessed by the improvement in IPSS, QoL, and Q_max_ using binary logistic regression analysis. The result was further verified by ROC analysis with a larger AUC in MBOOI. Additionally, to a certain extent, although some preoperative factors are related to surgical outcomes, such as P_ves_, P_det_Q_max_, P_abd_, BOOI, IPSS-t, QoL, and Q_max_, they are significantly less effective than MBOOI. Particularly, contrary to the findings of the previous study, an additional finding is that a significant correlation between TZI and surgical efficacy was not observed [26]. In addition, related studies have shown that the surgical effect of TURP is similar to that of green-light laser photo-selective vaporization of the prostate (PVP) and holmium laser enucleation of the prostate (HoLEP), and the PVP and HoLEP were not significantly better than the former [27–29]. Of course, A larger resected prostate tissue weight that is present after enucleation techniques, however, similar efficacy has been reported for enucleation techniques and TURP in the treatment of BPH. One RCT comparing holmium laser enucleation of the prostate (HoLEP) with TURP in BPH patients who completed the 7-yr follow-up found that the functional long-term results of HoLEP were comparable with TURP [28]. Therefore, we boldly assume that MBOOI can be applied to predict the surgical efficacy of enucleation techniques, but there is no relevant research at present, and more studies are needed to further clarify.

To the best of our knowledge, this is the first study to investigate the correlation between MBOOI and efficacy of TURP, and the results confirmed that MBOOI may be a potential candidate that can be used to predict surgical outcomes. The pathophysiology of male LUTS/BPH is highly complex and multifactorial, and the disease and efficacy are unlikely to be determined by a single factor [30]. For more optimal diagnosis and treatment of BPH, our task is to continuously explore the pathophysiological mechanism and determine more valuable indicators. This study provides new directions and ideas for this purpose.

There are several limitations to this study. First of all, one limitation of our implementation is that this is a retrospective study, and compared with non-invasive examination such as ultrasound, PFS as invasive examination may bring the risk of trauma to the patient. However, as the gold standard for diagnosing BOO, its status in urology is irreplaceable. We routinely completed this examination before TURP surgery, and the surgical efficacy was confirmed by reexamination 3 months after surgery. This study was retrospective and did not cause additional trauma to patients. In addition, some parameters that may influence the surgical outcome reported in the previous studies were not evaluated, such as intravesical prostatic protrusion, prostatic urethral angulation and ultrasonic estimation of bladder weight, detrusor wall thickness, and resistive index, et al. [31–34]. Further studies should carry out to compare the value of those parameters and MBOOI in predicting surgical efficacy. Thirdly, although Park et al. did not find a correlation between the resected prostate tissue ratio and surgical efficacy, there was no insufficient evidence to support this [35]. For example, Milonas et al. found that the volume of resected tissue was an important factor influencing the degree of symptom improvement [36]. Although the relationship between resected prostate tissue weight and surgical efficacy was not considered in our study, we tried to achieve completeness of resection intraoperatively. Additionally, resected prostate tissue weight is closely related to TZV, and our study shows that TZV has little effect in predicting surgical efficacy. Therefore, it is unlikely to radically change our study results. Finally, additional studies with larger samples are needed to further elucidate the relationship and mechanism between MBOOI and abdominal pressure with BPH and the surgical effect.

## Conclusions

Although both MBOOI and BOOI can predict the urinary symptoms in men with LUTS/BPH to a certain extent, however, there was a stronger correlation between MBOOI and LUTS. In addition, both AUC of MBOOI and BOOI for surgical efficacy was between 0.70 and 0.8, but MBOOI was slightly higher than that of BOOI. Meanwhile, our study indicates that MBOOI is significantly associated with improvements in IPSS, QoL, and Qmax after TURP. In general, these findings suggest that MBOOI may have greater potential than BOOI for evaluating disease and predicting surgical efficacy in patients with LUTS/BPH. Further research could quite beneficial to explain the role of MBOOI in the progression of disease and surgical prognosis in men with LUTS/BPH.

## Data Availability

The data used in the analysis are not publically available due to data protection, but anonymised data can be made available from corresponding author upon reasonable request.

## Abbreviations

BPH: Benign prostatic hyperplasia
BPO: Benign prostatic obstruction
BPE: Benign prostatic enlargement
LUTS: Low urinary tract symptom
QoL: Quality of Life
PFSs: Pressure-flow studies
BOO: Bladder outlet obstruction
BOOI: Bladder outlet obstruction index
Qmax: Maximum urine flow rate
MBOOI: Modified bladder outlet obstruction index
TURP: Transurethral resection of the prostate
PSA: Prostate-specific antigen
TRUS: Transrectal ultrasonograpy
TPV: Total prostate volume
TZV: Transitional zone volume
TZI: Transitional zone index
IPSS-s: Storage IPSS
IPPS-t: Total IPSS
IPSS-v: Voiding IPSS
IPSS-p: Post-micturitional IPSS
PVR: Post void residual urine volume
P_ves_: Intra-vesical pressure
P_abd_: Abdominal pressure
P_det_Q_max_: Detrusor pressure at maximum urine flow rate
ROC: Receiver operating characteristic
AUC: Area under the curve
PVP: Green-light laser photo-selective vaporization of the prostate
HoLEP: Holmium laser enucleation of the prostate

## References

[1] Cao N, Lu Q, Si J, Wang X, Ni J, Chen L, Gu B, Hu B (2017). The characteristics of transitional zone in prostate growth with age. Urology.

[2] Zhao J, Zhao Z, Song J, Ji Z, Tian Y (2011). The diagnostic accuracy and lower cutoff value of three methods for quantifying urethral resistance in men. Urol Int.

[3] Nitti VW (2015). Pressure flow urodynamic studies: the gold standard for diagnosing bladder outlet obstruction. Rev Urol.

[4] Tian Y, Su ZY, Liu DY, Yang B, Liu HM, Lei J, Luo GH, Sun ZL, Sun F, Xia SJ (2020). The clinical application value study of bladder outlet obstruction index on benign prostate hyperplasia. Nat J Androl.

[5] Han JH, Yu HS, Lee JY, Kim J, Kang DH, Kwon JK, Choi YD, Cho KS (2015). Simple modification of the bladder outlet obstruction index for better prediction of endoscopically-proven prostatic obstruction: a preliminary study. PLoS ONE.

[6] Oelke M, Bachmann A, Descazeaud A, Emberton M, Gravas S, Michel MC, N’dow J, Nordling J, de la Rosette JJ (2013). EAU guidelines on the treatment and follow-up of non-neurogenic male lower urinary tract symptoms including benign prostatic obstruction. Eur Urol.

[7] van Venrooij GE, van Melick HH, Eckhardt MD, Boon TA (2008). Diagnostic and predictive value of voiding diary data versus prostate volume, maximal free urinary flow rate, and Abrams-Griffiths number in men with lower urinary tract symptoms suggestive of benign prostatic hyperplasia. Urology.

[8] Seki N, Takei M, Yamaguchi A, Naito S (2006). Analysis of prognostic factors regarding the outcome after a transurethral resection for symptomatic benign prostatic enlargement. Neurourol Urodyn.

[9] Aarnink RG, de la Rosette JJ, Debruyne FM, Wijkstra H (1996). Formula-derived prostate volume determination. Eur Urol.

[10] Abrams PH, Griffiths DJ (1979). The assessment of prostatic obstruction from urodynamic measurements and from residual urine. Br J Urol.

[11] Homma Y, Kawabe K, Tsukamoto T, Yamaguchi O, Okada K, Aso Y, Watanabe H, Okajima E, Kumazawa J, Yamaguchi T, Ohashi Y (1996). Estimate criteria for efficacy of treatment in benign prostatic hyperplasia. Int J Urol.

[12] Griffiths D, Höfner K, van Mastrigt R, Rollema HJ, Spångberg A, Gleason D (1997). Standardization of terminology of lower urinary tract function: pressure-flow studies of voiding, urethral resistance, and urethral obstruction. Neurourol Urodyn.

[13] Joshi HN, De Jong IJ, Karmacharya RM, Shrestha B, Shrestha R (2014). Outcomes of transurethral resection of the prostate in benign prostatic hyperplasia comparing prostate size of more than 80 grams to prostate size less than 80 grams. Kathmandu Univ Med J (KUMJ).

[14] Kumar A, Vasudeva P, Kumar N, Nanda B, Jha SK, Mohanty N (2013). A prospective randomized comparative study of monopolar and bipolar transurethral resection of the prostate and photoselective vaporization of the prostate in patients who present with benign prostatic obstruction: a single center experience. J Endourol.

[15] Rhodes T, Girman CJ, Jacobsen SJ, Roberts RO, Guess HA, Lieber MM (1999). Longitudinal prostate growth rates during 5 years in randomly selected community men 40 to 79 years old. J Urol.

[16] Sarma AV, Jaffe CA, Schottenfeld D, Dunn R, Montie JE, Cooney KA, Wei JT (2002). Insulin-like growth factor-1, insulin-like growth factor binding protein-3, and body mass index: clinical correlates of prostate volume among Black men. Urology.

[17] Park JS, Koo KC, Kim HK, Chung BH, Lee KS (2019). Impact of metabolic syndrome-related factors on the development of benign prostatic hyperplasia and lower urinary tract symptoms in Asian population. Medicine.

[18] Kanik EA, Erdem E, Abidinoglu D, Acar D, Akbay E, Ulusoy E (2004). Can the outcome of transurethral resection of the prostate be predicted preoperatively?. Urology.

[19] Huang T, Yu YJ, Qi J, Xu D, Duan LJ, Ding J, Zhu YP (2015). Establishment and value assessment of efficacy prediction model about transurethral prostatectomy. Int J Urol.

[20] Oh MM, Kim JW, Kim JJ, du Moon G (2012). Is there a correlation between the outcome of transurethral resection of prostate and preoperative degree of bladder outlet obstruction?. Asian J Androl.

[21] Min DS, Cho HJ, Kang JY, Yoo TK, Cho JM (2013). Effect of transurethral resection of the prostate based on the degree of obstruction seen in urodynamic study. Korean J Urol.

[22] Han DH, Jeong YS, Choo MS, Lee KS (2008). The efficacy of transurethral resection of the prostate in the patients with weak bladder contractility index. Urology.

[23] Kim M, Jeong CW, Oh SJ (2017). Diagnostic value of urodynamic bladder outlet obstruction to select patients for transurethral surgery of the prostate: systematic review and meta-analysis. PLoS ONE.

[24] Sekido N (2012). Bladder contractility and urethral resistance relation: what does a pressure flow study tell us?. Int J Urol.

[25] Mijailovich SM, Sullivan MP, Yalla SV, Venegas JG (2004). Theoretical analysis of the effects of viscous losses and abdominal straining on urinary outlet function. Neurourol Urodyn.

[26] Milonas D, Saferis V, Jievaltas M (2008). Transition zone index and bothersomeness of voiding symptoms as predictors of early unfavorable outcomes after transurethral resection of prostate. Urol Int.

[27] Thomas JA, Tubaro A, Barber N, d'Ancona F, Muir G, Witzsch U, Grimm MO, Benejam J, Stolzenburg JU, Riddick A, Pahernik S, Roelink H, Ameye F, Saussine C, Bruyère F, Loidl W, Larner T, Gogoi NK, Hindley R, Muschter R, Thorpe A, Shrotri N, Graham S, Hamann M, Miller K, Schostak M, Capitán C, Knispel H, Bachmann A (2016). A multicenter randomized noninferiority trial comparing GreenLight-XPS laser vaporization of the prostate and transurethral resection of the prostate for the treatment of benign prostatic obstruction: two-yr outcomes of the GOLIATH study. Eur Urol.

[28] Gilling PJ, Wilson LC, King CJ, Westenberg AM, Frampton CM, Fraundorfer MR (2012). Long-term results of a randomized trial comparing holmium laser enucleation of the prostate and transurethral resection of the prostate: results at 7 years. BJU Int.

[29] Zhou Y, Xue B, Mohammad NA, Chen D, Sun X, Yang J, Dai G (2016). Greenlight high-performance system (HPS) 120-W laser vaporization versus transurethral resection of the prostate for the treatment of benign prostatic hyperplasia: a meta-analysis of the published results of randomized controlled trials. Lasers Med Sci.

[30] Wasson JH, Reda DJ, Bruskewitz RC, Elinson J, Keller AM, Henderson WG (1995). A comparison of transurethral surgery with watchful waiting for moderate symptoms of benign prostatic hyperplasia. The veterans affairs cooperative study group on transurethral resection of the prostate. N Engl J Med.

[31] Lee JW, Ryu JH, Yoo TK, Byun SS, Jeong YJ, Jung TY (2012). Relationship between intravesical prostatic protrusion and postoperative outcomes in patients with benign prostatic hyperplasia. Korean J Urol.

[32] Shim M, Bang WJ, Oh CY, Lee YS, Cho JS (2020). Correlation between prostatic urethral angulation and symptomatic improvement after surgery in patients with lower urinary tract symptoms according to prostate size. World J Urol.

[33] Huang T, Qi J, Yu YJ, Xu D, Jiao Y, Kang J, Chen YQ, Zhu YK (2012). Predictive value of resistive index, detrusor wall thickness and ultrasound estimated bladder weight regarding the outcome after transurethral prostatectomy for patients with lower urinary tract symptoms suggestive of benign prostatic obstruction. Int J Urol.

[34] Shinbo H, Kurita Y, Nakanishi T, Imanishi T, Otsuka A, Furuse H, Mugiya S, Ozono S (2010). Resistive index: a newly identified predictor of outcome of transurethral prostatectomy in patients with benign prostatic hyperplasia. Urology.

[35] Park HK, Paick SH, Lho YS, Jun KK, Kim HG (2012). Effect of the ratio of resected tissue in comparison with the prostate transitional zone volume on voiding function improvement after transurethral resection of prostate. Urology.

[36] Milonas D, Verikaite J, Jievaltas M (2015). The effect of complete transurethral resection of the prostate on symptoms, quality of life, and voiding function improvement. Cent Eur J Urol.

